# Current biological and pharmacological updates on *Tinospora cordifolia*

**DOI:** 10.17179/excli2024-7170

**Published:** 2024-05-21

**Authors:** Shubham Kumar, Murtaza M. Tambuwala, Yachana Mishra, Vijay Mishra

**Affiliations:** 1 School of Pharmaceutical Sciences, Lovely Professional University, Phagwara (Punjab)-144411, India; 2 College of Pharmacy, Ras Al Khaimah Medical and Health Sciences University, Ras Al Khaimah, United Arab Emirates; 3 School of Bioengineering and Biosciences, Lovely Professional University, Phagwara (Punjab)-144411, India

## ⁯⁯

**Figure 1:**
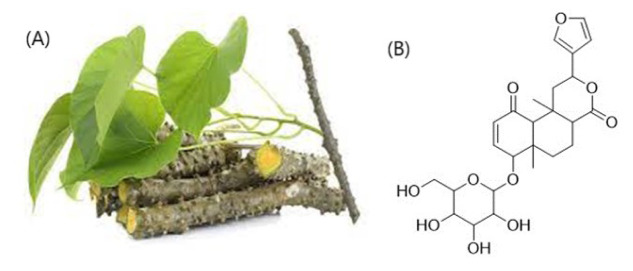
(A) Plant part of *T. Cordifolia* and (B) Chemical structure of *Tinosporaside*

Guduchi, scientifically known as *Tinospora cordifolia*, is one of the non-controversial and extensively used herbs in Ayurvedic medicine belonging to the family *Menispermaceae* (Sharma, 2017[17]). In the Ayurvedic healthcare system, guduchi is considered precious for its curative properties. The plant's numerous chemical contents, which are dispersed throughout its various plant sections, and each has unique medicinal properties, are responsible for its pharmacological actions. Glycosides, diterpenoid lactones, sesquiterpenoids, steroids, phenolic and aliphatic substances, essential oils, multiple kinds of fatty acids, polysaccharides, and tinosporaside (Figure 1(Fig. 1)) are among these chemical components (Ninama et al., 2022[9]). Only a few systematic reviews have been published highlighting the possible advantages of the plant, however, the majority of preclinical and clinical evidence demonstrated the wound healing, diabetes, hepatic, anti-toxic, anti-stress, inflammatory, and additional bioactive properties of *T. cordifolia*. As there is a scarcity of studies reflecting the therapeutic role of *T. cordifolia*, the present letter highlights the beneficial effects of *T. cordifolia* in the treatment of different diseases. Additionally, it gives researchers a direction for future research and suggests ways to improve the safety and effectiveness of *T. cordifolia* in treating a variety of diseases and other uses (Table 1(Tab. 1); References in Table 1: Ambalavanan et al., 2021[1]; Balkrishna et al., 2023[2]; Bhandari et al., 2022[3]; Gupta et al., 2018[4]; Kamboj et al., 2023[5]; Kumar et al., 2022[6]; Maya et al., 2022[7]; Mishra et al., 2023[8]; Philip et al., 2018[10]; Prakash Kumar et al., 2011[11]; Prasad and Chauhan, 2019[12]; Pushp et. al., 2013[13]; Salve et al., 2015[14]; Sarkar et al., 2023[15]; Sharma et al., 2019[16]; Singh et al., 2020[18]; Tiwari et al., 2014[19]; Yow et al., 2023[20]).

EXCLI Journal 2024;23:811-815 – ISSN 1611-2156
Received: March 12, 2024, accepted: April 05, 2024, published: May 21, 2024

| Therapeutic benefits | Key finding | Reference |
|---|---|---|
| Anti-oxidant and hepato-protective activity | Pushp et al. examined the antioxidant activity of *T. cordifolia* methanolic extract, demonstrating dose-dependent benefits in scavenging radicals, preventing lipid peroxidation, and lessening the toxicity of cyclophosphamide in mice. Antioxidant qualities of the extract were seen with increased glutathione (GSH) levels. This finding highlights the health benefits of *T. cordifolia* as a naturally occurring source of antioxidants. | Pushp et. al., 2013 |
| | The ethanolic extract of *T. cordifolia* demonstrated antioxidant properties in a dose-dependent manner by preventing lipid peroxidation, free radicals, and cyclophosphamide (CP) sensitivity. Kumar et al. revealed that plant extract strengthened the body's natural antioxidant defense by raising GSH levels. Histopathology evidence supported the protective effects of ethanol stem extract against CP-induced liver damage by lowering lipid peroxidation, raising GSH, superoxide dismutase (SOD), and catalase activities, and enhancing liver function. | Kumar et al., 2022 |
| | Balkrishna et al. highlighted *T. Cordifolia*'s robust antioxidant properties within a specific dosage range. Given individual differences, consulting healthcare providers for supplement dosages is recommended. For enhanced health and antioxidant benefits, a daily intake of 500-1000 mg is suggested. | Balkrishna et al., 2023 |
| Anti-infective activity | Prasad and Chauhan evaluated the antibacterial activity of *T. cordifolia* methanolic and ethanolic extracts against a range of bacteria and fungi. The methanol extract showed inhibitory areas of 9 mm over *E. coli* and other bacteria, whereas the zones were smaller (0.08-0.25 mm) for other solvents. With regards to antibacterial effectiveness, extracts from plant parts fared better than ethanol extracts. These findings demonstrated a promising anti-infective activity of methanolic extract against a variety of microbes. | Prasad and Chauhan, 2019 |
| | Gupta et al. evaluated an increase in pro-inflammatory cytokines, nitric oxide synthase 2 (NOS2), nitric oxide (NO) production, major histocompatibility complex class II (MHC-II), and CD-86 expression, all of which suggested classical macrophage activation. Besides triggering the mitogen-activated protein kinase (MAPK) pathways i.e. p38, extracellular signal-regulated kinase (ERK), and c-Jun amino (N)-terminal kinase (JNK) in *Mycobacterium tuberculosis* (Mtb)-infected macrophages, it also increased the generation of cytokines, NOS-2, MHC-II, and CD-86. Administration of polysaccharide rich extract (PRE) reduced the survival of drug-resistant Mtb within macrophages, a phenomenon that was largely attributed to PRE-mediated NO production. The result demonstrated how NO stimulation caused intracellular Mtb to be eliminated while also activating classical macrophages. | Gupta et al., 2018 |
| Antidiabetic activity | Mishra et al. stated that Tinosporaside, the key ingredient of *T. cordifolia*, showed tremendous potential in the treatment of diabetes and glucose utilization. *In vitro* studies demonstrated that it, like insulin, increased glucose absorption in skeletal muscle cells. Tinosporaside promoted glucose tolerance, decreased blood glucose levels, and improved insulin sensitivity in diabetic rats. It activated key glucose transport pathways, making it a viable natural drug for hyperglycemia and impaired insulin management. | Mishra et al., 2023 |

EXCLI Journal 2024;23:811-815 – ISSN 1611-2156
Received: March 12, 2024, accepted: April 05, 2024, published: May 21, 2024

| Therapeutic benefits | Key finding | Reference |
|---|---|---|
| | Yow et al. showed that orally administered *T. cordifolia* lowered glucose levels in the blood of diabetic rats considerably. It also enhanced wound contraction and epithelialization, showing its utility in wound healing. It was found to have anti-inflammatory qualities and to enhance granulation tissue development, presumably by controlling blood glucose levels. However, the role of *T. cordifolia* in angiogenesis is unknown. Furthermore, it increased fibroblast proliferation and collagen deposition, which helped to strengthen the wound. | Yow et al., 2023 |
| Hypolipidemic/ anti-obesity effect | Singh et al. noted the ability of the herbal formulation to combat obesity and hypolipidemia in a variety of ways. It lowers plasma cholesterol via downregulating hepatic 3-hydroxy-3-methyl-glutaryl-coenzyme A reductase (HMGCR), which mimics the actions of statins. Lowering acetyl-CoA carboxylase (ACC) expression promotes lipid oxidation, whilst boosting peroxisome proliferator-activated receptor (PPAR) promotes better lipid profiles. Increased endothelial nitric oxide synthase (eNOS) expression stimulated NO generation and vasodilation, demonstrating its anti-obesity efficacy. Increased expression of adipose triglyceride lipase (ATGL) aids in lipolysis and adipose mobilization, underlining *T. cordifolia*'s promise in the treatment of obesity and the improvement of lipid profiles. | Singh et al., 2020 |
| | Bhandari et al. represented that *T. cordifolia* aqueous extract (TCP) efficiently regulated body weight, dyslipidemia, and obesity in middle-aged female rats. TCP addresses several metabolic markers, including dyslipidemia, liver problems, and oxidative stress. Notably, TCP enhances feed and calorie intake while decreasing body weight gain in regular chow-fed middle-aged rats, indicating its ability to regulate body weight. TCP influences the expression of several proteins involved in lipid metabolism, including AMP-activated protein kinase (AMPK), ACC, sterol regulatory element binding transcription factor 1 (SREBP1), fatty acid synthase (FAS), ATGL, and peroxisome proliferator activated receptors (PPARs), implying a multifaceted involvement in metabolic health. | Bhandari et al., 2022 |
| Anti-inflammatory activity | *T. cordifolia* stem extract inhibited cyclooxygenase-2 (COX-2) and lipoxygenase (LOX) enzymes in an *in vitro* study. This suggests that the extract may have beneficial effects for circumstances involving leukotriene-mediated pathophysiology, though COX-1 selectivity was not confirmed. | Prakash Kumar et al., 2011 |
| | Mice administered with *T. cordifolia* extract showed lower oxidative stress markers, enhanced antioxidant activity, and decreased immunoglobulin E (IgE) levels as well as eosinophil count and airway hyperresponsiveness. *T. cordifolia* therapy decreased pro-inflammatory cytokines and increased anti-inflammatory interferon gamma (IFN-γ). Furthermore, as shown by periodic acid-Schiff (PAS) staining and histopathology, it increased IκBα expression while decreasing COX-2, ICAM-1, inducible nitric oxide synthase (iNOS), and c-jun $NH_2$-terminal kinase (JNK) levels. | Tiwari et al., 2014 |

EXCLI Journal 2024;23:811-815 – ISSN 1611-2156
Received: March 12, 2024, accepted: April 05, 2024, published: May 21, 2024

| Therapeutic benefits | Key finding | Reference |
|---|---|---|
| | Philip et al. reported that *T. cordifolia* extract showed anti-inflammatory properties by blocking the production of genes that promote inflammation, including iNOS, COX-2, tumor necrosis factor alpha (TNF-α), interleukin 6 (IL-6), and interleukin1 beta (IL-1β). This result was linked to the control of NF-κB and activation of the p38 protein. Notably, the COX-1 gene was not significantly affected by the *T. cordifolia* extract. The extract successfully decreased edema in an *in vivo* inflammation framework similar to indomethacin. To completely comprehend the underlying mechanisms and develop viable treatments for illnesses involving dysregulated innate immunity, more research is required. | Philip et al., 2018 |
| Nephroprotective activity | Ambalavanan et al. investigated *T. cordifolia*-based polylactic acid nanoparticles (TC-PLA NPs) for their effects in a rat model. Treatment improved diabetic rat blood glucose levels and body weight, lowered urine volume and protein excretion, and improved kidney function, as measured by lower levels of protein, albumin, urea, blood urea nitrogen (BUN), and creatinine. Furthermore, TC-PLA NPs reduced the levels of pro-inflammatory cytokines IL-6 and TNF-α, inhibited the advanced glycation end product (AGE) and their receptor (RAGE) signaling pathway, and improved kidney histology. These data suggest the potential of TC-PLA NPs to manage diabetic nephropathy by lowering inflammation, oxidative stress, and fibrosis. | Ambalavanan et al., 2021 |
| | Sharma et al. and Maya et al. investigated the possible nephroprotective properties of *T. cordifolia* in a situation where gentamicin accumulates in renal cortex cells, causing mitochondrial dysfunction, oxidative stress, and free radical production. Daily intake of 200 mg/kg ethanolic *T. cordifolia* extract therapy significantly reduces kidney damage, implying potential nephroprotection via antioxidant regulation. | Sharma et al., 2019; Maya et al., 2022 |
| Medicinal value | Salve et al. observed that daily administration of 150-300 mg *T. cordifolia* herb extract increased physical performance, including peak acceleration, grip strength, distance, and $VO_2$ max, in a trial with healthy participants, indicating its potential benefits under stress. | Salve et al., 2015 |
| | Sarkar et al. highlighted *T. cordifolia's* extensive potential as a countermeasure against SARS-CoV-2. Boosting immunity is essential. Connected with the Keap1 Nrf2 pathway, it demonstrated antiviral and immunomodulatory characteristics. When Keap1 is released, the component that triggers antioxidant responses (ARE) is activated and Nrf2 is adversely regulated. Enhancing endogenous antioxidants requires Keap1. The application of molecular docking for COVID-19 suppression demonstrated the potential of *T. cordifolia* to reduce cytokine storms caused by COVID-19 and offering tailored treatment approaches. | Sarkar et al., 2023 |
| Pharmaceutical application | Kamboj et al. fermented the stem of *T. cordifolia* using particular nutrients to produce a beverage with increased levels of ethanol, total phenols, and oxidative capability. Several probiotic strains were added to it, with *Lactiplantibacillus pentosus* demonstrating the best safety and survival rate for human cells. The drink was an innovative and promising product since it demonstrated antimicrobial qualities and included a variety of phytochemicals with antibacterial, antioxidant, and anti-inflammatory potential. | Kamboj et al., 2023 |

*Funding*

None.

*Conflict of interest*

The authors declare no conflict of interest.

## Declaration

### Funding

None.

### Conflict of interest

The authors declare no conflict of interest. 

## Figures and Tables

**Table 1:**
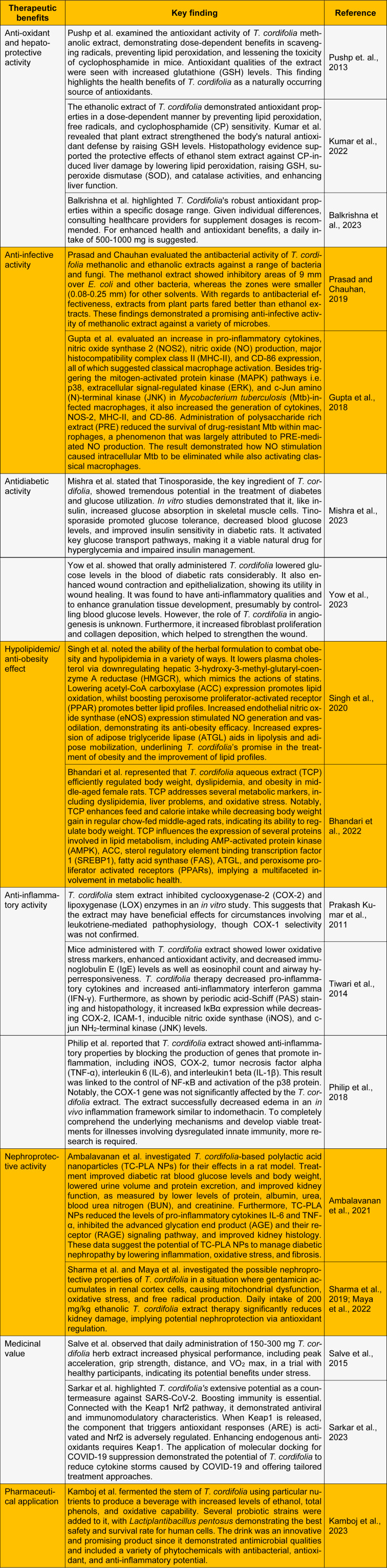
An update on the biological and pharmacological activities of *Tinospora cordifolia*
